# Income development of General Practitioners in eight European countries from 1975 to 2005

**DOI:** 10.1186/1472-6963-9-26

**Published:** 2009-02-09

**Authors:** Madelon W Kroneman, Jouke Van der Zee, Wim Groot

**Affiliations:** 1 NIVEL (Netherlands Institute of Health Services Research), PO Box 1568, 3500 BN Utrecht, The Netherlands; 2 Faculty of Health, Medicine and Life Sciences, Maastricht University, Maastricht, The Netherlands; 3 Faculty of Health Medicine and Life Sciences, Department of Health Organization, Policy and Economics, Maastricht University, Maastricht, The Netherlands

## Abstract

**Background:**

This study aims to gain insight into the international development of GP incomes over time through a comparative approach. The study is an extension of an earlier work (1975–1990, conducted in five yearly intervals). The research questions to be addressed in this paper are: 1) How can the remuneration system of GPs in a country be characterized? 2) How has the annual GP income developed over time in selected European countries? 3) What are the differences in GP incomes when differences in workload are taken into account? And 4) to what extent do remuneration systems, supply of GPs and gate-keeping contribute to the income position of GPs?

**Methods:**

Data were collected for Belgium, Denmark, Germany, Finland, France, the Netherlands, Sweden and the United Kingdom. Written sources, websites and country experts were consulted. The data for the years 1995 and 2000 were collected in 2004–2005. The data for 2005 were collected in 2006–2007.

**Results:**

During the period 1975–1990, the income of GPs, corrected for inflation, declined in all the countries under review. During the period 1995–2005, the situation changed significantly: The income of UK GPs rose to the very top position. Besides this, the gap between the top end (UK) and bottom end (Belgium) widened considerably. Practice costs form about 50% of total revenues, regardless of the absolute level of revenues. Analysis based on income per patient leads to a different ranking of countries compared to the ranking based on annual income. In countries with a relatively large supply of GPs, income per hour is lower. The type of remuneration appeared to have no effect on the financial position of the GPs in the countries in this study. In countries with a gate-keeping system the average GP income was systematically higher compared to countries with a direct-access system.

**Conclusion:**

There are substantial differences in the income of GPs among the countries included in this study. The discrepancy between countries has increased over time. The income of British GPs showed a marked increase from 2000 to 2005, due to the introduction of a new contract between the NHS and GPs.

## Background

The remuneration of doctors in general and General Practitioners in particular is an issue that has the attention of policy makers in many countries. During the past decades, in several countries health care reforms have affected the income of GPs (e.g. in France, the Netherlands and the UK). To what extent these developments are comparable with trends in other European countries is unknown. The aim of this paper is therefore to provide insight into the development of the incomes of General Practitioners (GPs) during the last decades in a number of European countries. This study is an update and extension of an earlier study by Delnoij [1], who provided an overview of the income situation of GPs in eight European countries for 1975–1990 A summary of the findings of the Delnoij study can be found in Annex 1 [see Additional file 1]. In the present study, we have added the years 1995, 2000 and 2005.

The theory of supplier-induced demand suggests that GPs can, at least to some extent, adjust their behaviour in order to secure their income. A precondition for this is that the remuneration system provides incentives for supplier-induced demand [2-4]. The possibilities for General Practitioners to generate an income are influenced by health care system characteristics and by market forces (competition among GPs and between GPs and other physicians). In the next paragraphs the effect of remuneration system, access to primary care and competition will be discussed.

The *type of remuneration *(salary, capitation fee or fee-for-service) provides different opportunities for increasing income. When physicians receive a salary, increasing the number of paid working hours can generate more income. However, working more than a certain number of hours (more than full-time) is often not compensated for. In systems where physicians receive a capitation fee (this is a fixed amount per year for each patient on the GP's list), physicians can boost their income by increasing the number of patients registered at their practice (if there is no fixed maximum number of listed patients). For GPs who are paid a fee-for-service, providing more services can generate a higher income. However, salaries or fees may be subject to budget caps and/or agreements among GPs and (governmental) health authorities or insurance companies. These agreements are often the result of negotiations between both parties and are valid for a fixed time period. There is evidence that (changes in) fee-for-service remuneration influences physician's behaviour [2,3,5-8]. Therefore, we assume that GPs who are paid a fee-for-service have more opportunities to increase their income and will thus have a higher income compared to their salaried and capitation fee paid colleagues.

*Competition among GPs *depends on the supply of GPs and their position compared to other health care providers. We assume that for countries with an ample supply of GPs, the relative income position of GPs will be less favourable compared to countries where GPs are relatively scarce. The bargaining position of GPs relative to other physicians (e.g. medical specialists) is strongly influenced by what is generally called the 'gate keeping' system: when patients need a referral from a GP for access to specialist care, GPs do not have to compete with medical specialists for patients [9,10]. We therefore assume that the income position of GPs in gate-keeping countries and in countries with relatively fewer GPs is better than in direct access countries and in countries where GPs are more abundant.

Delnoij [1] only compared the annual income of GPs. We believe that, since GP workload differs among countries [11], the results may differ if workload is taken into account. Thus besides comparing annual income, we included a comparison of income per hour worked and per patient attended to.

The research questions that will be answered in this paper are:

• How can the remuneration system of GPs in a country be characterized?

• How has the annual GP income developed over time in selected European countries?

• What are the differences in GP income when differences in workload are taken into account?

• To what extent do the remuneration system, the supply of GPs and gate-keeping contribute to the income situation of GPs?

## Methods

### Concepts and operationalizations

#### General Practitioner

The first concept concerns the question: What is a General Practitioner? Here we used a pragmatic approach; for each country we selected the type of physician that is indicated by the following terms (or their equivalent as translated into English): General Practitioner or Family Physician. As a result, in some countries physicians that may provide directly accessible and ambulatory medical care, for instance paediatricians, gynaecologists and internists, were excluded.

#### Income and types of remuneration

The *revenues *of GPs consist of the total remuneration received from practising their profession.

In countries where GPs are self-employed, *practice costs *are included in the fee structure. *Practice costs *are costs that are intrinsic to practising general medicine and have to be subtracted from the revenues of GPs. Practice costs roughly consist of the following components: housing, personnel, medical equipment, ICT, insurances and transportation. In this study, practice costs are included only when GPs themselves are responsible for these costs. In most countries there is no readily available statistic on practice costs. Accordingly, we used estimates provided by country experts, preferably well documented. In order to improve comparability among the countries, we will not include remuneration for out-of-hours care, when possible.

The *income *of GPs was calculated as revenue minus practice costs. For *salaried *GPs, the revenues are equal to their income, since the practice costs in these systems are normally the responsibility of the health care organization that employs the GPs. For GPs that receive *capitation fees*, the revenue is the (annual) fee for patients multiplied by the number of patients in a full-time general practice. Their income is this revenue minus practice costs. These capitation fees are sometimes differentiated according to certain criteria such as age or social deprivation. For GPs that receive *fee-for-service *remuneration, several types of service (e.g. office consultations, home visits, telephone consultation, vaccinations) may have different tariffs. The income is calculated as revenues from fees-for-service minus practice costs.

The raw revenue data are often provided in local currencies or Euros. In order to make figures comparable between countries, we converted the revenue data into Purchasing Power Parities (PPPs) [12]. PPPs can be defined as currency conversion rates that both convert to a common currency and equalize the purchasing power of different currencies. They eliminate the differences in price levels between countries in the process of converting economic indicators expressed in a national currency to an artificial common currency, called the Purchasing Power Standard (PPS), expressed in US dollars. We derived the PPPs from the World Development Indicator reports 1997 (Table 5.5), 2002 (Table 5.6) and 2006 (Table 4.14, PPP conversion rates for 2004) [13-15] (See Annex 1: Calculation of GP revenue per country [see Additional file 1]). When the available data were not from the exact year requested (but for instance from one year earlier or later), we corrected these data for inflation, using data from the OECD health data files. When practice costs were available for one year only, we estimated the practice costs for the requested years using the available figure corrected for inflation. To compare GP revenue over time, we adjusted the revenue figures by the Consumer Price Index, with the year 2000 as the reference year (source: OECD.Stat Extracts [16]).

#### Competition

For *GP-supply *we use the data provided by country experts. When these were not available, we used GP-density data from within the OECD health data files [17]. Other physicians that provide directly accessible ambulatory care (e.g. paediatricians, internists) were excluded. Information on whether a *gate-keeping *system exists or specialist care is directly accessible for patients is derived from the work of Kroneman *et al*. [9].

#### Workload

Workload was operationalized in two ways: firstly, by the number of hours worked per week. The exact number of hours worked by full-time GPs may differ per country. Where possible, the number of working hours per week was derived from information supplied by country experts. When this was not available we used the data provided by the study of Boerma *et al*. on GP task profiles (data from 1993) [11]. The income or profit per hour was calculated as follows: annual income or profit/((52-5 weeks)*working hours per week). We assumed 5 weeks holiday per year. Secondly, the number of patients attended to per full-time GP was determined. The income (or profit) per patient was calculated as annual income (or profit) divided by the number of patients served per full-time GP. The number of patients per full-time GP is not equal to the reciprocal of GP supply, since the first figure is based on full-time working GPs and the latter on all (including part-time working) GPs.

### Data collection

As this study is a replication and an extension of the study of Delnoij, the countries included are the same: Belgium, Denmark, Finland, France, Germany, the Netherlands, Sweden and the United Kingdom. The data from 1990 and before were derived from Delnoij [1]. For the information on the remuneration of GPs, we firstly consulted existing written sources (e.g. the European Observatory on Health Care Systems) and websites. When the data were either not available or language problems prevented interpretation, we consulted country experts. The country experts were either GPs, members of national GP-associations or medical associations or members of research institutes dealing with health care issues. When an expert was not able to provide the data requested, we asked whether he/she could provide the name of a person who could. We sent a tailor-made questionnaire to the experts, in which we included what was already known about the remuneration system, in order to make completion of the questionnaire as easy as possible. The final country descriptions were sent to the experts with the request to check for errors and wrong interpretations. For extensive country descriptions, see Annex 1: Calculation of GP revenue per country [see Additional file 1].

The source of the data may influence the results of this study: interest groups, like GP associations may tend to underestimate GP income and overestimate practice costs compared, for instance, to government bodies or insurance companies, since this can influence the bargaining position of both parties. For this reason, the source of the data is reported in Table 1.

**Table 1:** Data source and health care organization characteristics related to GP revenue.

	**Data sources**		**Health care organization characteristics**	
**Country**	**Revenue/income**	**Practice costs**	**Remuneration system**	**Gate-keeping**
Belgium	Interest group	Interest group	Fee-for-service	No
Denmark	Interest group and government agency	Interest group and government agency	Fee-for-service	Yes
Finland	WHO (2000) and independent organization	-	Mainly salary, some additional fee-for-service and capitation fee	Yes
France	Government agency	Tax office	Fee-for-service	No
Germany	Independent organization	Independent organization	Fee-for-service	No
Netherlands	Interest group	National statistics office (based on tax data)	Capitation and fee-for-service^1)^	Yes
Sweden	Interest group	-	Salary	No
U.K.	Independent organization (1995, 2000), government agency (2005)	Independent organization (1995, 2000), government agency (2005)	Capitation, since 2004 additional quality related payments	Yes

^1) ^The remuneration of GPs in The Netherlands is a combination of a capitation fee for the publicly insured patients (about 2/3 of the annual revenues) and a fee-for-service for the privately insured patients (about 1/3 of the total revenues). Since the share of fee-for-service is substantial, the Netherlands is characterized in the analyses as a fee-for-service country

#### Differences compared to Delnoij

For the sake of comparability, the same sources should ideally be used in our study as in Delnoij's. However, it was not always possible to use the same source, and sometimes more reliable data were available (for instance, the practice costs for France in our study were based on GP tax forms; in the study of Delnoij they were based on an estimate of 40% of the total revenues). The exact differences can be found in Annex 1: Calculation of GP revenue per country [see Additional file 1].

### Analyses

For the remuneration system and gate-keeping system, the countries were divided into two groups: fee-for-service payment and non-service related payments (capitation fees and salary). For access to care also two groups were formed: firstly, the gate-keeping system consisted of countries where patients have to go to a GP first (gate-keeping) and secondly countries where patients may go to specialists directly (direct access). The average income per year for each group was calculated and plotted over time. Due to the low number of observations (eight countries) statistical analyses were not applicable. For the workload, cross-sectional comparisons were made for the year 2000. The differences in ranking of the countries between annual income and income corrected for workload was evaluated using Spearman's rho. The relationship between GP-supply and income was evaluated using Pearson's correlation coefficient from the SPSS statistical package version 14.0.

## Results

### Country descriptions (situation for 1995, 2000 and 2005)

#### Belgium

The Belgian GP is remunerated on the basis of a fee-for-service system. Extra revenue is generated by having patients of 50 years and older on the GP's list (managing their medical record). These extra remunerations (so called *GMD-vergoedingen*: General Medical Record allowances) were introduced in 1999 and are included in the figures for 2000 and 2005.

#### Denmark

In Denmark, the GPs derive their revenue from a capitation fee, which makes up one third to half of their revenue, and from fees for services rendered (per consultation, examination, procedure etc.). In the period from 1995 to 2005 there was no change in the remuneration system but only small adjustments in the fees and the types of fees. However, the proportion of fees for services has gone up due to an increase in activities in GP practices.

#### Finland

In Finland general practitioners work in health centres. Two payment systems exist. Traditionally, payment is mainly based on a monthly salary with additional payments based on, among other factors, seniority and skills. Work that exceeds 37 hours a week is remunerated separately from the salary.

The second system is called the personal doctor system, which is a special remuneration formula determined by a basic salary (which can be only about 60–85% of the traditional monthly salary described in the paragraph above), that is supplemented by a payment per consultation (5–6 euro per consultation) for patients that consulted their GP less than three times in the previous year and a monthly "capitation" payment (1.60 – 1.95 euro per month) for the so-called frequent visitors – those who visited their GP more than three times in the previous year. This programme leads to higher total revenue compared to the traditional system, but the GP is not protected by the limit of 37 hours, as there is no formal working week, just a requirement to offer services on weekdays.

In Finland a small private sector exists, where physicians are paid on a fee-for-service basis. In 1999 only 8% of all doctors worked full time in private practice. These private sector GPs were excluded from our analysis.

#### France

General Practitioners in France are mainly paid on a fee-for-service basis. In 2005 the "*Médecin traitant*" (treating physician) was introduced. This physician keeps the medical record of the patient and makes referrals to other medical care. In March 2007, 82% of the insured population had chosen a *médecin traitant *[18], 99% of whom were GPs. Patients who don't register with a *médecin traitant*, or who visit a specialist without consulting their *médecin traitant*, receive lower reimbursement. Then treating physicians received, in addition to the usual fee-for-service, ~40 per year for each patient with a chronic disease who chose them as record keeper.

#### Germany

The payment of ambulatory physicians (both general practitioners and specialists) is a two-stage process. First, the public health insurance funds make total payments to physicians' associations in the form of negotiated capitation fees for each member (insured person) of the fund. These negotiated budgets are subsequently distributed among the members of the physicians' associations according to a Uniform Value Scale. This scale contains a list of all services that can be provided by physicians for remuneration within the statutory health insurance system. Each of these services is awarded a certain number of points. Physicians invoice their associations each quarter for the total number of points generated by the services rendered. The total negotiated budget is divided by total number of points. The monetary value of the points is then used to calculate the physicians' quarterly remuneration. From 1997 to 2003 the number of reimbursable points per patient was limited. In 2007 the remuneration system will have changed to a system with negotiated morbidity-oriented service volumes.

#### The Netherlands

Until 2006, the remuneration of Dutch GPs depended on the type of insurance held by the patient. For publicly insured patients, GPs received a capitation fee. Since 1995 the fee depended on the number of patients in the practice: the flat fee for the first 1,600 patients on the list was higher than for those above the 1,600. In 2000 and 2005 the capitation fee was differentiated by the patient's age [see Additional file 1]. For privately insured patients, a fee for service system existed, with fixed fees. Approximately 2/3 of the population was publicly insured and 1/3 privately [19]. For the purposes of this paper, our analysis of the Netherlands is based on this system.

#### Sweden

In Sweden, county councils are responsible for ambulatory health care provision. Payments to primary care centres are normally based on all-in budgets, and payment principles may vary among the county councils. Physicians are in salaried service at primary care centres. As such, they receive a monthly salary from the county councils. Since the mid 1990s, in some counties GPs receive an additional capitation fee for each patient, to increase their monthly income. In the last decades, the differentiation among the Swedish counties has increased and nowadays there exist as many health care systems as there are counties in Sweden, with all counties operating a different mix of salary, capitation and fee-for-service for GPs, which makes it impossible to determine "the" Swedish remuneration system.

#### United Kingdom

Before 2004, the revenues of physicians in the United Kingdom were determined by a basic allowance, supplemented by allowances based on factors including the number of listed patients, patient characteristics (age, chronic conditions, living in deprived areas) and some types of services rendered. There was a slight gradient in revenue due to seniority (depending on the number of years a GP is registered).

In 2004, the payment system changed from GP-based to practice-based, with an all-in budget. Payment was still based on characteristics of the patient list, but additional revenue could be earned when certain quality requirements were met. There were four domains for quality improvement: the clinical domain (with an emphasis on certain diseases), the organizational domain (including: information, communication, education and practice management), the additional services domain (cervical screening, child health surveillance, maternity services and contraceptive services) and finally the patient experience domain which consists of how services are provided and the involvement of patients in service development plans.

The type of remuneration system per country is summarized in Table 1.

### Developments in GP income over time

In Additional file 2], the average revenue, practice costs and income of GPs in the eight countries in our study are displayed in monetary terms.

**Figure 1:**
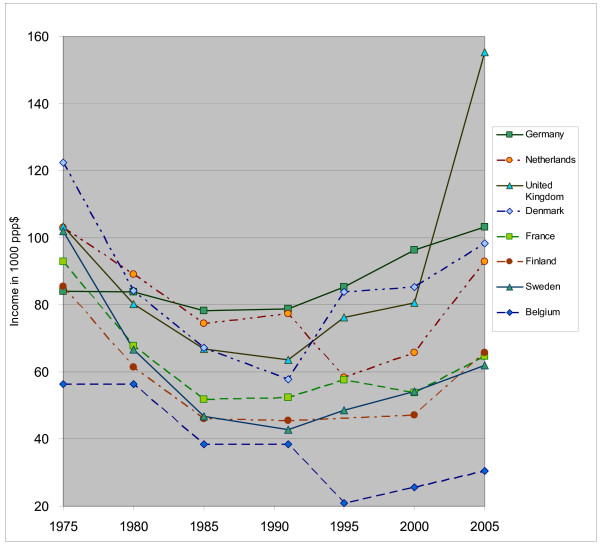
**Annual GP income over time in ppp$, corrected for inflation, index year = 2000**.

When we deflate GP income by the consumer price index (using OECD data on the consumer price index with base year 2000 = 100, see Table 2), we see that the income of GPs declined during the period from 1975 to 1985, then stabilized until the year 1995 and after that increased again, although real income in 2005 is still lower than in 1975 for most countries (except for the UK and Germany). Figure 1 reveals that the differences between the countries became larger in 2005 compared to the earlier years. GP income in Belgium is the lowest compared to the other countries, this difference becoming even more sizeable in recent years. GP income in the UK increased markedly from 2000 to 2005, turning the British GPs into the best paid doctors compared to their colleagues in the other countries (see Figure 1 and Table 2).

**Table 2:** Income of GPs over time, corrected for inflation, index year is 2000 = 100

**Country**	**1975^1)^**	**1980^1)^**	**1985^1)^**	**1990**	**1995**	**2000**	**2005**
Belgium	56,309	56,295	38,476	38,389	20,864	25,602	30,413
Denmark	122,355	84,239	67,283	57,747	83,782	85,362	98,249
Finland	85,485	61,433	46,132	45,566	-	47,213	65,801
France	92,876	67,729	51,827	52,401	57,670	53,889	64,607
Germany	84,048	83,897	78,192	78,723	85,342	96,325	103,158
Netherlands	102,988	89,120	74,360	77,305	58,267	65,842	92,945
Sweden	101,959	66,685	46,832	42,812	48,594	54,124	62,007
United Kingdom	103,297	80,289	66,864	63,624	76,278	80,580	155,360

^1) ^Figures based on Delnoij [1]

**Figure 2:**
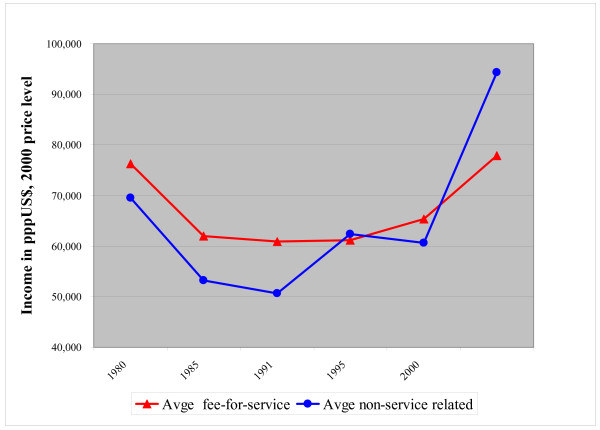
**Fee-for-service versus non-service related annual income (salary and capitation fee) over time in pppUS$, corrected for inflation (index year is 2000)^1)^**. ^1) ^The data for 1975–1990 are based on Delnoij [1].

The average inflation corrected annual income of GPs in countries with a salary or capitation system appeared to be lower than the incomes in fee-for-service payment systems until the beginning of the 1990s (see Figure 2). In 1995 and 2000 no difference was found between both remuneration systems. In 2005 the situation changed into a more favourable position for non-service related remuneration systems. This was again mainly due to the high rise in income for the UK GPs.

### Practice costs

In 2005, in the countries where GPs are independent professionals, the share of practice costs in total revenues was relatively similar between countries (varying from 46% to 61%, with an average of 52%), although in absolute terms (in pppUS$) the practice costs varied considerably. In the UK, practice costs in 2005 were about four times higher than in Belgium (see Table 2). During the 1995–2005 period, the average share of practice costs varied from 50% to 56%. Two outliers were found: Belgium at the top end, with over 60% for the period from 1995 to 2000 and the UK at the bottom end with 29% in 2000. When we look at the average practice costs per patient in pppUS$ in 2000, Germany ranks highest and UK lowest (see Table 3). Due to the large numbers of patients in Dutch GP practices, Dutch practice costs per patient are relatively low. From 2000 to 2005, the UK changed from having the lowest practice costs per patient to the highest practice costs per patient.

**Table 3:** Overview of practice costs (2000 and 2005) per patient in countries where GPs are independent entrepreneurs in pppUS$

**Country**	**Practice costs 2000**	**Number of patients per GP^1) ^2000**	**Practice costs per patient 2000**	**Practice costs 2005**	**Number of patients per GP^1) ^2005**	**Practice costs per patient 2005**
**Belgium**	46,709	860	54.31	51,941	860	60.40
**Denmark**	70,109	1311	53.48	98,541	1285	76.69
**France**	46,422	622	74.68	59,778	605	98.81
**Germany**	113,846	937	121.44	124,606	1027	121.33
**Netherlands**	94,709	2529	37.45	107,115	2529	42.35
**UK**	32,829	1600	20.52	216,545	1415	153.04

1)For Belgium, Denmark and The Netherlands, the number of patients per GP is based on the figures for a full-time GP from country specific sources [see Additional 1], for the other countries, the figures are derived from the OECD health data files 2006.

### GP income and workload

The annual income of GPs may differ among countries because of differences in workload (see Table 4). Workload can be expressed in either number of patients that have to be taken care of or working hours.

**Table 4:** Different income units (per year, per patient and per working hour) for 2000 (income data in pppUS$, sorted by income on annual basis)

**Country**	**Annual income 2000**	**Number of patients per GP^1)^**	**Income per patient**	**Working hours^2)^**	**Income per hour**	**GP-density**
Germany	96,325	937	102.75	60	31.03	1.1
Denmark	85,362	1311	65.11	43	38.53	0.9
UK	80,580	1600	50.37	58	26.72	0.6
Netherlands	65,842	2529	26.03	44	28.78	0.5
Sweden	54,124	1898	28.52	38	27.18	0.5
France	53,889	622	81.40	56	18.44	1.6
Finland	47,213	1384	34.11	38	23.89	0.7
Belgium	25,602	860	29.77	40	12.31	2.1

1) For Belgium, Denmark and the Netherlands, the number of patients per GP is based on the figures for a full-time GP as used in this study; for the other countries, the figures are derived from the OECD health data files 2006.

2) Working hours per week. For Belgium, Denmark, France, Germany, the Netherlands and the UK the figures are based on data collected in this study. For the UK, the figure includes being on-call [26]. For Finland and Sweden, the figures are based on the rather old study of Boerma (data collection in 1993) [27].

3) The remuneration of GPs in the Netherlands is a combination of a capitation fee for the publicly insured patients (about 2/3 of the annual revenues) and a fee-for-service for the privately insured patients (about 1/3 of the total revenues). Since the share of fee-for-service is substantial, we characterized the Netherlands as a fee-for-service country.

The income *per patient *for 2000 showed a top position for Germany and France with about 80–100 pppUS$ per patient. The UK and Denmark captured a middle position (about 50 pppUS$ per patient). All other countries had a much lower income per patient, around 25–35 pppUS$. Compared to the ranking of countries by annual income, the situation of France and the Netherlands was remarkable. France ranked much higher when income was expressed in income per patient and the Netherlands much lower compared to their annual income position. Statistical analysis showed that there was no significant relationship between the rankings of the countries for annual income and income per patient (Spearman's rho = 0.45, p = 0.26).

The highest *income per hour *in 2000 was earned in Denmark (slightly more than 38 pppUS$) followed by Germany (31 pppUS$ per patient). The lowest income per hour was earned in France and Belgium (less than 20 pppUS$). In all other countries GPs earned between 20 and 30 pppUS$ per hour. The ranking of countries by income per hour and income per year were significantly correlated (Spearman's rho = 0.88, p = 0.004).

### Competition

GP-supply in 2000 (see Table 4) showed a strong negative relationship with income per working hour (Pearson's r = -0.79, p = 0.02), but no relationship with GP annual income (Pearson's r = -0.48, p = 0.23) and income per patient (Pearson's r = 0.24, p = 0.57).

**Figure 3:**
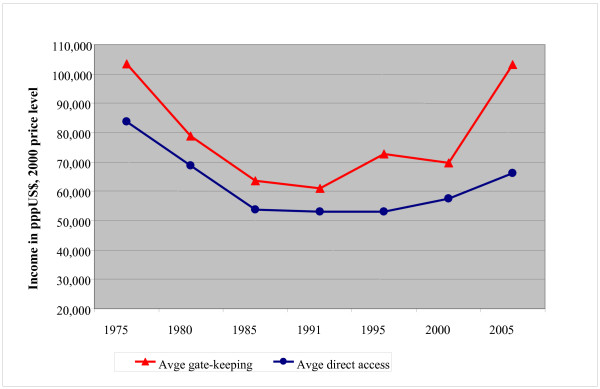
**The average annual income of GPs in gate-keeping versus direct access countries over time, corrected for inflation (index year = 2000)^1)^**. ^1) ^The data for 1975–1990 are based on Delnoij [1].

Over time, GPs in gate-keeping countries showed a higher income on average compared to their colleagues in direct access countries, (see Figure 3).

## Discussion

### Income over time

The annual income of GPs varied substantially among the eight countries in this study, both over time and among countries. Corrected for inflation, the purchasing power of GPs declined during the period from 1975 until 1990, which is probably the result of cost containment policies in most countries during that time. From 1995 onwards, in most countries the income position of GPs improved, with the greatest increase taking place in the UK. An unexpected finding was that income differences between the countries have increased in the past decade. The introduction of the new remuneration system in the UK in 2004 had a striking effect on the income of British GPs, resulting in the position of best paid GPs in 2005. Belgian GPs remained the lowest paid GPs over time. The low income of Belgian GPs may be an artefact of the calculation method, in which we used estimates based on a paper provided by the Belgian Association of GPs. However, an external audit in 2002 in Belgium produced similar results [20], supporting our findings.

The increase in income differences over time is somewhat surprising, as it might have been expected that improved opportunities for job mobility within the EU would have had an equalizing effect on income. The findings suggest that institutional factors are more important than market competition in explaining GP income differences between countries. The income differences may also reflect differences in status and importance attached to GP services, as well as differences in the bargaining power of GPs between countries.

Independent GPs appeared to spend on average 50% of their revenues on practice costs, regardless of the absolute level of the revenues. This implies that GPs with a higher income also generate higher practice costs.

We cannot conclude that one specific remuneration system generated higher incomes than another throughout the years under review.

### Workload

Limiting oneself to the comparison of annual income neglects differences in workload between countries. The fact that the workload of GPs varies internationally has been established in the study of Boerma [11]. We discovered that the country rank order of GP income per patient and income per working hour differed slightly from the annual income ranking, although the difference was not statistically significant. However, since the currently available information on workload is rather outdated, the influence of workload on income should be assessed in future research, in combination with research on workload differences.

### Competition

We argued that a large supply of GPs would lead to a weaker income position. The supply of GPs showed no relationship with income per year, which is in contrast to earlier findings [1,21]. However, in countries with a relatively large number of GPs, income per hour was lower. Accordingly, it seemed that GPs have to work more hours when there are fewer patients per GP available to obtain a similar level of annual income.

We also argued that GPs in a gate-keeping system have a better market position compared to GPs in a direct access system, because the former do not have to compete for patients with their specialized colleagues. The results of this study seem to support this hypothesis, since over time the average income of GPs in gate-keeping countries is higher compared to the average income of their colleagues in countries with direct access to secondary care.

### Limitations of the study

This study has several limitations, which will be discussed below.

#### Limited data

Our study was based on the data of eight countries, which is of course a very low number. As a result, outlier positions may strongly influence the relationships that have been studied. We believe, however, that this study is relevant, since it is, to our knowledge, the only longitudinal study of GP income, where health care system characteristics have been taken into account.

#### Revenues, costs and income

In most countries, GP revenue is not the subject of routine monitoring. Ideally, income figures should be compiled from information on tax declarations. When this is not available, the GP income may be based on estimates. These figures can be biased, depending on the interest of the organization that provides the estimate. Organized interest groups of GPs may provide a lower estimate for income and a higher estimate for costs compared to organizations that have to pay the remuneration (government bodies or health insurance organizations). As an example, we show differences in the estimates of practice costs of Dutch GPs. In the Netherlands, three different sources were available for estimating practice costs: The estimate by the body that establishes the fee rates for GPs (CTG-Zaio [22]) is much lower (60% of the total income) than the estimate calculated on behalf of the Dutch National Association of General Practitioners (LHV [23]; 93% of the total revenues in our calculations). This may also explain the high practice costs for Belgium, because the source used here was an estimate established by a group representing the interest of GPs. Furthermore, the difference in share of practice costs for UK GPs over time could also be explained by this phenomenon. The practice costs in the remuneration system before 2004 were estimated by the Review Body on Doctors' and Dentists' Remuneration. Although this is an independent organization, due to the lack of reliable data on practice costs, a rather old study on practice costs was used to obtain their estimates. The review body argued that practice costs had decreased since 1990 because of the introduction of computers [24], resulting in the relatively low share of practice costs (29%) in 2000. The figure for 2005 for the UK was based on tax declarations and will probably be more realistic.

In this study we tried to harmonize income figures by attempting to compare full-time GP incomes without out-of-hour compensation. However, the data were not always available in this format. For France and the UK, for instance, the income data relate to average GP income and for Germany and France, out-of-hours compensation is included. The share of out-of-hours compensation varies among countries. For Germany, out-of-hour compensation is estimated to be about 1.4% of the income generated from publicly insured patients [25]; in the Netherlands, this income component will increase the revenues by 7% on average.

#### Practice cost components

Some components of practice costs may contain a hidden income component. Sometimes practice costs are reimbursed on the basis of actual expenditure, whereas other costs are reimbursed according to estimates and are payable, regardless of whether these costs have been made or not and independently of the real expenditure. In the latter case, this reimbursement may (partly) result in extra income when this type of practice costs is lower or even absent. In the UK for instance, the compensation for ICT-equipment is rather generous and may exceed actual expenditure. The expenditure for social security payments may also differ. In the UK, the pension premium is fully compensated for. In the Netherlands and Germany, GPs have to pay these costs from their annual income.

#### Comparison over time

The slight lack of continuity in trends between the figures of Delnoij (1975–1990) and this study (1995–2005) may reflect real changes in GP income. However, it is also plausible that differences in data collection and calculation methods account for a major part of the lack of continuity. This is especially the case for Belgium. In Delnoij's study practice costs were estimated at 30% of total income, whereas in our estimates practice costs account for 60–70% of total income. When we changed the estimate of the practice cost in our calculations to 30%, Belgium remained at the lower end, slightly above Finland.

#### Other methodological issues

An important limitation of the study is the small number of countries included, due to which the strong increase in income in the United Kingdom in 2005 had a relatively large influence on the results.

In international comparative research, the comparability of the data is always a point of discussion. In our study this is also a relevant issue. For instance, working hours for some countries are based on relatively old data (1993) and the number of GPs in a country is not always easily established. Sometimes non-active GPs are included in figures; in other countries, GPs may not be counted as separate group of physicians, in which case an estimate must be made. Another problem is the exclusion of paediatricians and internists from the calculation of GP supply. In countries where paediatricians and/or internists are directly accessible for primary care services as well, the estimate of GP supply may be too low (e.g. in Germany).

## Conclusion

To summarize, we conclude that there are substantial differences in income of GPs over time and among the countries included in this study. Income differences have also increased in the last decade. The remuneration system appears to have no clear influence on the income of GPs. A larger supply of GPs in a country, was associated with a lower income per hour. GPs in gate-keeping systems appear to have a higher average income over time compared to their colleagues in countries with direct access systems. Further research is necessary on workload differences among GPs (including part-time work) and on practice costs: what elements are included and what are the determinants of the extent of these costs.

### Changes after 2005

Future trends after 2005 may depend on new changes in remuneration systems.

In the Netherlands, the system of privately and publicly insured persons was abolished in January 2006 and a new system with a basic insurance for all was introduced. At the same time, the remuneration system for GPs was changed to a basic capitation fee, differentiated by age and deprivation area and supplemented by a fee-for-service system for each consultation. This current system, which is not included in the income calculations of this study, is expected to influence Dutch GP incomes. In the UK, the rise in income will probably not continue, as payment levels were frozen in the 2006–2007 contract. A new survey in 2010 should reveal whether the increasing trend since 1995, shown in Figure 1, will continue or not.

## Competing interests

The authors declare that they have no competing interests.

## Authors' contributions

MK drafted the manuscript, carried out the analyses and collected the data. JZ initiated the study, participated in the design and helped to draft the manuscript. WG conducted a critical review of the manuscript and helped with the analyses. All authors read and approved the final manuscript.

## Pre-publication history

The pre-publication history for this paper can be accessed here:



## Supplementary Material

Additional file 1 **Annex 1.** Calculation of GP revenue per country revised. Click here for file

Additional file 2 **Overview of annual GP income for the years 1995, 2000 and 2005 in pppUS$^1^), countries in alphabetical order.** Click here for file
