# Supplementary material for: Income development of General Practitioners in eight European countries from 1975 to 2005
Source: BMC Health Serv Res. 2009 Feb 9;9:26. doi: 10.1186/1472-6963-9-26 (PMC2670288; doi:10.1186/1472-6963-9-26)
Supplement: Additional file 1 — Annex 1. Calculation of GP revenue per country revised. [file 1472-6963-9-26-S1.doc]

# Annex 1. Calculation of GP revenue per country

### SHORT DESCRIPTION OF THE STUDY OF DELNOIJ, 1975-1991 [1]

Delnoij published in 1994 as part of her thesis a chapter on GP income development from 1975-1991. The study included eight countries: Belgium, Denmark, Germany, Finland, France, The Netherlands, Sweden and the United Kingdom. Delnoij concentrated on two factors that can theoretically affect physician income: economic factors (supply, demand, competition), and political factors (the bargaining power of organized medicine). The relationships were expected to exist with regard to cross-national differences in the *level* of the GPs’ income as well as the *development* of their income. It was also expected that the development of GP income between 1975 and 1990 had been conditioned by the room the payment and the negotiation system left GPs as a group to increase their income through increasing the volume of care: if GPs had the room to increase their income through increasing volume, the *development* of their income between 1975 and 1990 did not depend on the development as regards the number of inhabitants per GP.

Delnoij formulated three hypotheses concerning the level of GP income and the development of their income:

1. The relationship between the number of inhabitants per GP and GP income is positive, such that (a): GP income is higher in countries where the number of inhabitants per GP is higher, and (b) over time GP income increases more (or decreases less) in countries where the number of inhabitants per GP increases more (or decreases less).
2. The relationship between GPs’ collective bargaining power and GP income is positive, such that: (a) GP income is higher in countries where GPs collectively hold a stronger position, and (b) over time GP income increases more (or decreases less) in countries where GPs collectively hold a stronger position.
3. If GPs can increase their income by increasing the quantity of services provided, the development of their income depends less on the development as regards the number of inhabitants per GP (p. 108, [1])

Delnoij used the following data: GP income was defined as average gross revenue from general practice minus practice expenses, expressed in US$ppp. For GP power the position in the UK, Demark and The Netherlands were evaluated as strong, because in these countries, GPs (1) are independent, self-employed contractors with a national health service or health insurance organization, 92) serve as gatekeepers to specialists and hospital care, 93) have a monopoly on the provision of primary medical care, and (4) are represented by their own GP association in income negotiations. Possibilities to obtain higher income through increased volume were established to be moderate in Belgium and France and absent in the other countries.

The results do not provide evidence strong enough for these hypotheses to be rejected, though they do not strongly corroborate them either. GP income is higher where GP density is lower, except for countries with a strongly hospital-dominated health care like Sweden and Finland (until 1980). Gps incomes compared with per capita GDP have decreased since 1975. The decrease is, however, due to economic factors, namely the increase in GP supply. However, if GPs can increase their income by providing more services, which is the case in Belgium and France, they seem to be able to partly offset the negative consequences of increased physician supply. Evaluation of the development of GP income further showed that British GPs did remarkably well between 1975 and 1990. Dutch and German GPs, on the contrary, experienced a serious decrease in their relative incomes, that is, compared with per capita GDP, although they still rank number two and number one on the list of highest incomes in US$ppp.

The results lead to the rejection of hypothesis 1 for countries with a strongly hospital-dominated health care, such as Sweden and Finland (until 1980). In other countries, however, GP income is higher where GP density is lower. Hypothesis 2 must be rejected as far as the *level* of income is concerned. The GPs’ position is, however, a significant factor in explaining the *development* of income. In general GP income compared with per capita GDP has decreased since 1975, but this decrease has been smaller for GPs who collectively hold a strong political position. Apart from political factors, decrease of GP incomes can be explained by economic factors, namely the increase in GP supply. Finally, if GPs can increase their incomes by providing more services, which is the case in Belgium and France, they can partly offset the negative consequences of increased physician supply. The latter finding suggests that hypothesis 2 cannot be rejected. It should, however, be noted that the number of observations in this study is low. Income data could be analyzed for eight countries only.

### TECHNICAL NOTES

The income per country will be provided in pppUS$. When selected sources resulted in different income estimates, we used the lowest estimate. In the overview below, the income in pppUS$ that is used in the paper is printed bold.

### Conversion rates

The following conversion factors were used throughout the document:

Table 1. Power Purchasing Parities (ppp) and Euro conversion rates

|  | Local currency to pppUS$ | | | Euro to local currency |
| --- | --- | --- | --- | --- |
|  | Based on ‘old’ currency | | Based on Euro |
| Country | 1995 | 2000 | 2005 |  |
| Belgium | 35.6 | 35.6 | 0.9 | 40.3399 |
| Denmark | 8.3 | 8.9 | 8.4 | 7.4538 |
| Finland | 5.7 | 6.1 | 1 | 5.94573 |
| France | 6.1 | 6.5 | 0.9 | 6.55957 |
| Germany | 1.9 | 1.9 | 0.9 | 1.95583 |
| Netherlands | 2.0 | 2.1 | 0.9 | 2.20371 |
| Sweden | 8.8 | 9.7 | 9.6 | 8.4452 |
| United Kingdom | 0.6 | 0.7 | 0.6 | 0.6095 |

( Table 1 continued)

Sources:

| ppp 1995: | World development indicators 1997, The World Bank, Washington, DC, USA, Table 5.5 (for local currencies) | |
| --- | --- | --- |
|  | Germany: 1995 is estimated from the figures for 2000 and 1990, which are both 1.9 | |
| ppp 2000: | World development indicators 2002, The World Bank, Washington, DC, USA, Table 5.6 (for local currencies) | |
| ppp 2005 | World development indicators 2006, The World Bank, Washington DC, USA, Table 4.14 (figures for 2004) | |
| Euro conversion rate (euro to local currency) for euro-countries: | | <http://www.euro.ecb.int/en/section/conversion.html> |
| Euro conversion rate: for other countries: | | [http://www.statistics.dnb.nl](http://www.statistics.dnb.nl/) |

Where data were provided in Euro’s in the original source in 1995 and 2000, the data were converted into local currency and then to ppp$US.

### Differences with Delnoij:

Belgium:

Delnoij used an estimate for practice expenses that on average amounted 25-30% of the total income. We used a more detailed foundation of the practice expenses, based on a policy-paper of the Belgian Association of Physicians (*ASGB: Algemeen Syndicaat van Geneeskundigen van België)* concerning the income of Belgian GPs [2], information on the internet and email correspondence with ASGB.

Finland:

We used the income figure expressed in the European Observatory series [3]. Delnoij used national statistics, but they will probably be the same source as the data form the European Observatory series. For 2005 we used information provided by the country expert.

France:

In our study, the practice expenses for 1995, 2000 and 2005 were based on tax-figures. In Delnoij’s study, the estimate for practice expenses was 40% of the total income. In our study, this estimate is 46%.

The Netherlands:

Delnoij estimated the income of Dutch GPs based on the capitation fee for publicly insured patients. Revenue from privately insured patients was assumed to be equal to the full capitation fee paid for publicly insured patients. In our estimate, we used workload as the basis for calculation for privately insured patients. Workload was measured in the second Dutch National Study on General Practice [4,5]. The estimate for practice expenses used by Delnoij was based on the figure used in the tariff negotiations. We used an estimate based on real expenses, which were derived from tax-forms [6].

Sweden:

Delnoij used an estimate of the ‘average’ income based on calculations of the Federation of County Councils. We also used an estimate of the ‘average’ income, but the figure was derived from the questionnaire filled out by the Swedish Medical Association.

United Kingdom:

For 1995 and 2000, the same type of sources was used compared with Delnoij. Due to a change of payment system, for 2005 other sources were consulted

### COUNTRY DESCRIPTIONS

### Belgium:

*Type of remuneration*

The Belgium GP is remunerated on the basis of a fee-for-service system.

*Establishment of tariffs or salary*

The fees result from negotiations within the Committee of mutualities (these are sick funds) and physicians. The fees require endorsement of the Minister of Social Affairs. Besides this, a majority of physicians should agree with the fees. The fees are normally set for two years. Physicians are allowed to charge more than the negotiated fee. However, they risk suspension from practice when charging under the set level [7].

*Sources and calculation of revenue and income*

The income figures for Belgium were based on a policy-paper of the Belgian Association of Physicians (*ASGB: Algemeen Syndicaat van Geneeskundigen van België)* concerning the income of Belgian GPs [2] and email correspondence with ASGB.

In the ASGB paper, the calculation of the income is based on a working day of 8 hours, 5 days a week, 11 month per year. It is assumed that 1.5 hours per day is spent on non-patient bound activities, like schooling, meetings, and literature. This leaves 6.5 hours for patient contacts, from which 70% are assumed to be contacts at the GPs office and 30% home visits. The average duration of an office contact is 20 minutes, of a home visit 30 minutes. This results in 20 home visits and 68 office consultations in a week. On a monthly basis this sums up to 80 home visits and 272 office consultations. Yearly (based on 11 months) this is 880 home visits and 2992 consultations. Based on an average of 4.5 contacts per person per year, a full-time GP will serve a patient population of 860 persons. Extra income is generated by having patients of 50 years and older on the GP’s list (managing their medical record). The assumption is that 25% of the patients are older than 50 years and 60% of these patients will have appointed the GP as their medical record holder. This results in 126 extra remunerations (so called *GMD-vergoedingen*: General Medical Record allowances). These GMD-allowances were introduced in 1999 and are included in the figures for 2000 and 2005.

An important remark regarding the above workload calculations is the following: Having 860 persons as patient population is an improbable outcome of the calculation due to the fact that there are on average 479 inhabitants per GP available in Belgium (10,251,250 inhabitants and 21,415 GPs in 2000 (source: OECD Health data files 2005)).

Table 2. Estimation of income of full-time Belgian GP.

| **In Euro** |  | **1995** | |  | **2000** | |  | **2005** |  |
| --- | --- | --- | --- | --- | --- | --- | --- | --- | --- |
| **Service** | **Number per year (2002)**  **(A)** | **Fee**  **(B)** | | **Income**  **(A*B)** | **Fee**  **(C)** | | **Income**  **(A*C)** | **Fee**  **(D)** | **Income**  **(A*D)** |
| Home visits | 880 | 16.63 | | € 14,634 | 18.74 | | € 16,491 | 29.00 | € 25,520 |
| Consultations | 2992 | 13.63 | | € 40,781 | 15.29 | | € 45,748 | 16.41 | € 49,099 |
| GMD-vergoeding | 126 |  | | € - | 12.51 | | € 1,576 | 20.00 | € 2,520 |
| **Total Income + practice costs** | |  | | € 55,415 |  | | € 63,815 |  | € 77,139 |
| Inflation correction |  |  | | 92% |  | | 98% |  | 111% |
| Source |  |  | OECD 2005 | |  | OECD 2005 | |  | Eurostat |
| **Practice costs** |  |  | | € 38,459 |  | | € 41,221 |  | € 46,747 |
| **Income excl. practice costs** | |  | | € 16,956 |  | | € 22,594 |  | € 30,392 |
| **In pppUS$** |  |  | |  |  | |  |  |  |
| **Income + practice costs** | |  | | **62,794** |  | | **72,312** |  | **85,709** |
| **Practice costs** | |  | | **43,580** |  | | **46,709** |  | **51,940** |
| **Income excl. practice costs** | |  | | **19,214** |  | | **25,602** |  | **33,768** |

The practice costs were also estimated in the ASGB discussion paper and include office costs (building, electricity, telephone, furnishing, information technology, insurances), personnel (practice assistant and cleaner), and the costs of a middle class car [2]. These data were based on the year 2001. For the years 1995 and 2005 the same data were used, corrected for inflation.

### Denmark

*Type of remuneration*

In Denmark, GPs derive their income from a capitation fee, which makes up one third to half of their income, and from fees for services rendered (per consultation, examination, operation etc.).

*Establishment of tariffs or salary*

The fees are negotiated between the Organisation of General Practitioners (PLO) and the NHSS (National Health Security System) committee, which is run by the Association of County Councils. Costs for housing and staff are included in the fee structure.

*Detailed description of (changes in) remuneration system*

The Danish GPs serve as gatekeepers (although a very small proportion (1,7%) of the population fits into another scheme, where they are free to choose the physician of their choice. These people have to pay for all services except hospital treatment). There is no differentiation towards patient’s age in fees. Until 1995/96 children under the age of 16 were not individually registered with the GP but along with their parents (mother). If the child had a consultation, the service was registered under the parent’s civil registration number but marked with a “child mark”. After 1995 all children were also registered individually. This had no special impact on the earnings but made it possible to identify each registered person in a practice (e.g. consultations per registered patient). In the period from 1995 to 2005 there has been no change in the remuneration system but there have been small adjustments in the fees and the types of fees. However, the proportion of fee for services has increased due to higher activity in practice.

*Sources and calculation of revenue and income*

For the income of Danish GPs, two sources were available:

One source of data originated from the *Amtsrådsforeningen* (ARF). From the ARF data for 1996, 2000 and 2004 were available. The ARF supplied data on total GP income and practice costs. GPs have to pay your own pensions and social contributions and those for their staff. In Denmark in 2002-2004, health preventive/promoting consultations were introduced. In 2005 GPs provided 130,000 of these consultations. If they are in included in the ordinary surgery consultations the mean is: 3.57. According to the ARF figures, in 1996 Danish GPs received on average 1,223,314 DK (143.953 pppUS$) and in 2000 1,383,696 DK (155.471 pppUS$) on an annual basis, including practice costs. Approximately half of this amount is spent on practice expenses.

Another source of data was the *Praktiserende Lægers Organisation* (PLO), the Danish Organisation of Practicing Physicians. From the PLO, data for 1997 and 2003 were available. The PLO provided data on fee rates and number of services per inhabitant. We used the lowest of both revenue estimations in our overview.

Table 3. Estimation of income of Danish GP based on data of ARF

|  | **1996** | **1995** |  | **2000** |  | **2004** | **2005** | |
| --- | --- | --- | --- | --- | --- | --- | --- | --- |
|  | **DC** | **Corrected for inflation** | **ppp$** | **DC** | **ppp$** | **DC** | **Corrected for inflation** | **ppp$** |
|  |  | 98% |  |  |  |  | 103% |  |
| **Income with practice costs** | 1,225,314 | 1,194,814 | **143,953** | 1,383,696 | **155,471** | 1,694,021 | 1,737,418 | **206,835** |
| **Income without practice costs** | 636,173 | 620,338 | **74,739** | 759,726 | **85,362** | 886,953 | 909,675 | **108,295** |
| **Practice costs** | 589,141 | 574,476 | **69,214** | 623,970 | **70,109** | 807,068 | 827,743 | **98,541** |

Table 4. Estimation of income of Danish GP, based on data of PLO

| **Income component** | **Service rates in Danish Crowns (DC)** | | **# services**  **/inhabitant** | |  | **Number of inhab /GP^1)^** | | | **Total income from services** | | |
| --- | --- | --- | --- | --- | --- | --- | --- | --- | --- | --- | --- |
|  | **1997** | **2003** | **1997** | **2003** | **2004^1)^** | **1995** | **2000** | **2004** | **1997** | **2003** | **2004** |
|  | (A) | (B) | (C) | (D) | (E) | (F) | (G) | (H) | (A*C*F) | (B*D*G) | (B*E*H) |
| Consultation | 92.26 | 101.77 | 3.8 | 3.9 | 3.55 | 1241 | 1311 | 1540 | 435,080 | 520,340 | 556,377 |
| Telephone consultation | 21.21 | 23.4 | 2.58 | 3.08 | 2.99 | 1241 | 1311 | 1540 | 67,910 | 94,486 | 107,748 |
| Home visit (average) | 151.09 | 166.65 | 0.28 | 0.14 | 0.16 | 1241 | 1311 | 1540 | 52,501 | 30,587 | 41,063 |
| Total capitation fee | 809,026 | 1,088,717 |  |  |  |  |  |  | 809,026 | 1,088,717 | 1,088,717 |
|  |  |  |  |  |  |  |  |  | **1995** | **2000** | **2005** |
| Corrected for inflation: | | Income from services (DC) including practice costs | | | | | | | 530,187 | 608,880 | 797,979 |
|  |  | Income from capitation including practice costs | | | | | | | 772,174 | 1,027,092 | 1,145,911 |
|  |  | **Total income (DC)** | | | | | | | 1,302,361 | 1,635,972 | 1,943,890 |

1. Data from ARF, because these were available for the exact year or most nearby of the years requested.

Table 4a. Inflation correction factors

| 1995 vs 1997 | 95% |
| --- | --- |
| 2000 vs 2003 | 94% |
| 2005 vs 2003 | 105% |

The income estimates based on the data of the PLO are 9% higher for 1995,18% higher for 2000, and9% higher for 2005 compared to the ARF.

### Finland

*Type of remuneration*

The Finnish GPs receive mainly a salary. There are two remuneration systems, one comprising of a salary and one comprising of a salary with additional fee-for-services and capitation fees.

*Establishment of tariffs or salary*

The salary for physicians is negotiated among the physicians’ union and the Commission for Local Authority Employers.

*Detailed description of (changes in) the remuneration system*

In Finland general practitioners work in health centres. A health centre is a functional unit that provides primary curative, preventive and public health services to its surrounding population. Health centres are owned by (one or more) municipalities. Since early 1990's, there have been two distinct alternatives for GP payment. The “traditional” payment is mainly based on a monthly salary with additional payments based on amongst others seniority and skills. Work that exceeds 37 hours a week is compensated apart from the salary.

In health care centres where the personal doctor system has been introduced there is a special remuneration formula, which is based on a basic salary (which can be only about 60-85 % of that described above), on consultations and procedures and on a specially calculated "capitation" payment. The consultation fees are paid for patients who have seen a doctor less than three times during the previous year. Once this limit is exceeded, the patient will no longer generate consultation fees, but will be added to the group of frequent visitors. The number of such patients is the basis of a monthly capitation payment. This programme leads to clearly higher total income, but the doctor is not protected by the limit of 37 hours, as there is no formal working hours, just a requirement to offer services on weekdays.

In the private sector physicians are paid fee-for-service. In 1999 only 8% of all doctors worked full time in private practice. This system is not included in this study.

Later, in the 2000’s, the clear divide between the two public systems has been obscured by all kind of variants, which the local health centres / municipalities created to attract doctors or retain them. Exact figures on these extras are not available.

*Sources and calculation of income*

For Finland for 2000, income data were available in the European Observatory on Health Systems, in the Finland edition [3]. Presented is the income for a full-time GP. For 2005 we received data from STAKES, National Research and Development Centre for Welfare and Health, with income figures for November 2006. The monthly average salary was then 5,888 euros, which amounts to 70,656 euros per year. The inflation correction for 2005 was 98.7%, based on price index data from the OECD health data files 2007. For 1995 no data were available.

The basic salary of a GP was in 2000 on average Fmk 15,000 (47.213 pppUS$) per month and the total salary, including payments for being on duty and other extras was Fmk 24,000. [3]

The health centre pays practice costs. The doctors get paid for the use of their own car for work purposes at a national annually agreed rate (0.43 euros per kilometre). The health centre is responsible for getting locums, although often the volume of service is reduced for the summer vacation season.

Table 5. Estimation of income of Finnish GP

|  | **1995** |  | **2000** |  | *2006* |  | **2005** |  |
| --- | --- | --- | --- | --- | --- | --- | --- | --- |
|  | **Fmk** | **pppUS$** | **Fmk** | **pppUS$** | *Euro* | *pppUS$* | **Euro** | **pppUS$** |
| Income incl practice costs | - | - | 288,000 | **47,213** | *70,656* | *70,656* | 69,725 | **69,725** |
| Practice costs | - | - | - | **-** | *-* |  |  |  |
| Income excl practice costs | - | - | 288,000 | **47,213** | *70,656* | *70,656* | 69,725 | **69,725** |

### France

*Type of remuneration*

General Practitioners in France are mainly paid fee-for-service.

*Establishment of tariffs or salary*

For the tariffs, all GPs are bound to national agreements between physicians (GPs and specialists together) unions and the health insurance funds that are valid for four to five years. The current agreement was signed in January 2005 and involves annual expenditure targets (in terms of annual growth rates) on sick leave payments and drug prescriptions (antibiotics, statins, etc.). General increases in tariffs are dependent on whether these targets are met. French GPs are obliged to charge the official fees. A small number of GPs (so called sector 2 physicians) may charge higher fees; they represent 13 % of all French GPs.

*Detailed description of (changes in) the remuneration system*

In 1995 and 2000, services were assigned a key letter and a coefficient. Each key letter corresponded with a certain group of services of similar nature. The key letter is assigned a certain value and the coefficient accounts for the relative importance of the procedure within its group. The charge for a procedure is calculated by multiplying the value of the key letter with the coefficient. In 2005 a new system was implemented: the services only have a code and a tariff*.* A GP visit is coded “C”, for “consultation”, and the value of C is currently 21 € (22 € in July 07 and probably 23 € after mid-2008).

In 2001 supplementary payment was introduced for GPs that registered patients (system named “Médecin référent”). For each registered patient an extra fee of €46 was introduced [8]. However in 2003, only 1% of the patients and 10% of the GPs joined the program, and this program was discontinued. In 2005 a new system, called “*Médecin traitant*” was introduced in 2005. In March 2007 82% of the insured population had chosen a *médecin traitant* [9], 99 % of whom being GPs. Patients who don’t register with a *médecin traitant*, or who visit a specialist without being addressed by their *médecin traitant*, get less reimbursed. Moreover, GPs receive 40€ per year for each patient with a chronic disease (“ALD”, for “Affection de Longue Durée”) who chose them as *médecin traitant*.

*Sources and calculation of revenue and income*

The data for France come from the Ministry of Health, statistical office. The sources are two publications that are available on-line. Data on workload and fees was available from the *Assurance Maladie, caisse nationale* (National Sickness Fund). The practice costs are average practice costs for both single-handed practices and group practices and were available from the *Direction Générale des Impôts* (Tax Department at the Ministry of Finance). The practice costs include housing, furnishing, staff, transportation, communication (telephone and computer), insurance, professional taxes (but not income taxes) and mandatory social contributions (e.g. pensions, health, invalidity). The mandatory social contributions account for 13% of total revenue (resulting in € 11,500 for 1995 and € 13,000 for 2000). The practice costs do not include replacement of GPs in case of holidays. The locum tenants are excluded from the income figures as well. As a result, the costs for locum tenants in France are not included in this overview. In 2005, the income from out-of-hours services represented an average of 5,000 euros per GP. This income is included in the overview in Table 6.

Table 6. Estimation of income of French GP

|  | **1995** | | **2000** | | **2005** | |
| --- | --- | --- | --- | --- | --- | --- |
|  | **Euro** | **pppUS$** | **Euro** | **pppUS$** | **Euro** | **pppUS$** |
| Income including practice costs | 88,500 | **95,168** | 99,400 | **100,311** | 117,700 | **130,778** |
| Practice costs^1)^ | 38,000 | **40,863** | 46,000 | **46,422** | 53,800 | **59,778** |
| Income excl. practice costs | 50,500 | **54,305** | 53,400 | **53,889** | 63,900 | **71,000** |

^1)^ Source: email correspondence with *CNAMTS*

### Germany

*Type of remuneration*

The German remuneration system can be characterized as a fee-for-service system.

*Establishment of tariffs or salary*

Budget negotiations will take place between physicians’ associations and sickness funds.

*Detailed description of (changes in) the remuneration system*

The payment of ambulatory physicians (both general practitioners and specialists) is a two-stage process. Firstly, the sickness funds make total payments to physicians’ associations in the form of negotiated capitation fees for each member (insured person) of the fund. These negotiated budgets are distributed among the members of the physicians’ associations according to a Uniform Value Scale. This scale lists all services that can be provided by physicians for remuneration within the statutory health insurance system. Each of these services is given a certain value. Physicians invoice their associations each quarter for the total number of points. The total negotiated budget is divided by total number of points. The monetary value of the points is then used to calculate the physicians’ quarterly remuneration. From 1997 to 2003 the number of reimbursable points per patient was limited. In 2002 there were 116,065 Social Health Insurance (SHI)-affiliated physicians, from which 51% practised as GP. Approximately 88% of the German population is covered by the social health insurance [10]. In 2007 the remuneration system will change in a system with negotiated morbidity-oriented service volumes.

*Sources and calculation of revenue and income*

In Germany the Central Institute for GP care (*Zentralinstitut für die kassenärztliche Versorgung* (ZI)) published on a regular basis the Cost Structure Analyses (*Kostenstrukturanalyse*). The data were based on tax-forms of a representative sample of the national registry of physicians . The reimbursement included practice expenses, which consisted of approximately 57% of the total remuneration (57.5% in West-Germany and 56.1% in East-Germany in 2001). Practice costs consisted of costs for personnel, office, medical equipment and disposables. German GPs worked more than 55 hours per week. This is more than a person who was working in full-time salaried civil service in Germany [11].

The above-mentioned figures relate only to self-employed general practitioners working in their own practice in the outpatient care system in Germany.

The total medical revenue contains payments for social health insured people covered by social health insurance and some private payments from private patients.

Salaried physicians are not included in the above-mentioned figures. In Germany mainly hospital-based physicians are salaried.

Table 7. Estimation of income of German GP based on data of Central Research Institute of Ambulatory care in Germany

|  | **1995*** |  | **2000*** |  | **2004** |  |
| --- | --- | --- | --- | --- | --- | --- |
|  | **Euro^1)^** | **ppp$** | **Euro^1)^** | **ppp$** | **Euro** | **ppp$** |
| Total medical revenue | 184,710 | **190,138** | 204,172 | **210,171** | 211,657 | **236,300** |
| - Social health insurance | 155,771 |  | 171,032 |  | 176,296 |  |
| - Private patients | 28,939 |  | 33,140 |  | 35,361 |  |
| Total operating costs | 106,834 | **109,973** | 110,596 | **113,846** | 111,611 | **124,606** |
| Income before taxes | 77,876 | **80,164** | 93,575 | **96,325** | 100,046 | **111,694** |
|  |  |  |  |  |  |  |

^1)^ Only West Germany

### The Netherlands

Type of remuneration

In the Netherlands, until 2006, the remuneration system consisted of a mix of capitation fee (for publicly insured patients) and a fee-for-service system (for privately insured patients).

*Establishment of tariffs or salary*

Both types of remuneration were negotiated with the Central Body of Tariffs in Health Care (*College Tarieven Gezondheidszorg, Zorgautoriteit (CTG))*. In the Netherlands GP income is based on a so-called normative practice (*normpraktijk*), which theoretically consists of 2350 patients. In real, income may differ from this norm.

*Detailed description of (changes in) the remuneration system*

The remuneration of the GP was until 2006 dependent on the type of insurance of the patient. For publicly insured patients, GPs received a capitation fee. In 1995 the fee was dependent on the number of patients in the practice (for all patients up to 1600 registered patients, GPs received a fee of 55.79 Euro (59 pppUS$), for all patients above 1600, the fee was 31.61 Euro (33 pppUS$). In 2000 and 2005 the capitation fee was differentiated by age of the patient. For privately insured patients, a fee for service system existed, with fixed fees. Approximately 70% of the population was publicly insured and 30% privately.

For patients living in deprived areas higher fees existed in 2000 and 2005, but this is not included in these calculations. The assumption was that patients from deprived areas have a larger health care consumption, due to which a GP can serve fewer patients. To stimulate GPs to work in these areas, the higher fee was introduced, in order to compensate for the ‘extra’ work per patient.

Since January 2006, the system with privately and publicly insured persons was abolished and a new system with a basic insurance for all was introduced. The remuneration system for GPs was changed in basic capitation fee, differentiated towards age and deprivation area and completed with a fee-for-service system for each consultation. This system is not included in the income calculations of this study.

*Sources and calculation of revenue and income*

For the Netherlands, the GP income was estimated from the fee structure (provided by the National GP Association (*Landelijke Huisartsenvereninging (LHV)*) and the Central Body on Fees in Health Care (*Centraal Orgaan Tarieven Gezondheidszorg/Zorgautoriteit in oprichting (CTG-Zaio)*) and number of patients per FTE and workload available from the Second National Survey on General Practice (*Nationale studie naar ziekte en verrichtingen in de huisartsenpraktijk)* carried out in 2001 [12,13]. Additional income can be obtained by administering influenza vaccinations for the population that is recommended for vaccination. For each vaccination the GP received fl 17.75 (8.45 pppUS$) in 2000 and Euro 9.02 in 2005. The fee for influenza vaccinations was available from the national foundation for the influenza vaccination campaign (Stichting Nationaal Programma Grieppreventie (SNGP)). Influenza vaccination uptake was available from the Netherlands Information Network of General Practice (*Landelijk Informatienetwerk Huisartsenzorg)[14]*. Not included in the income figures were compensation for out-of-hour duty (approximately 300 hours per year, increasing the income with € 14,843 per year) and extra income from special services like cervical swab, minor surgery, ECG’s, third party medical examinations and out-of-hours services.

Practice costs were not readily available and several sources revealed different levels of practice costs. The estimate of practice costs of the Dutch GP Association (*Landelijke Huisartsen Vereniging (LHV))* provided the highest estimate with 134.000 euro in 2000 [15]. The estimate of the Central Tariff Body (*CTG)* that was used for the establishment of the fees the lowest. For 2000 (based on figures of 2006, corrected for inflation) the amount was 87.000 euro [16]. The difference between both figures is largely the result of higher estimates for personnel costs (approximately 13.500 euro higher) and deputy service (locum tenant) (approximately 9.000 euro) by the *LHV*. An attempt to estimate practice costs from tax-data was made by the Central Bureau for Statistics based on data of 227 practices in 2001 [6]. Based on these calculations, the practice costs for a solo practice in 2000 would be 90.000 euro (94.700 pppUS$). We used this latter figure for the calculation of practice costs.

Table 9. Fee structure for Dutch GP

| **Fee structure** | **1995** | **2000** | **2005** |  |
| --- | --- | --- | --- | --- |
| Public health insurance | EURO | EURO | EURO |  |
| Capitation <1600 patients (A) | 55.79 |  |  |  |
| Capitation > 1600 patients (B) | 31.61 |  |  |  |
| Capitation <65 yrs (C) |  | 63.49 | 77.00 |  |
| Capitation > 65 yrs (D) |  | 74.92 | 90.80 |  |
|  |  |  |  |  |
| Private insurance |  |  |  |  |
| Consultation (E) | 15.88 | 17.97 | 24.80 |  |
| Home visit (F) | 23.82 | 26.95 | 37.20 |  |
| Telephone consultation (G) | 7.94 | 8.98 | 12.40 |  |
|  |  |  |  |  |
| Influenza vaccination per shot for high risk patient | | 8.05 | 9.02 |  |

In the calculations the ‘other contacts’ and ‘unknown’ contacts from Table 10 are included in the calculations for the same fee as telephone consultations.

Table 10. Workload of Dutch GP1)

| **Workload** | **2001** |
| --- | --- |
| Patients per fte (H) | 2529 |
| 65+ (I) | 13.0% |
| Sickness fund patients (J) | 64.7% |
| Consults/patient (K) | 1.936 |
| Home visits/patient (L) | 0.221 |
| Phone contacts/patient (M) | 0.286 |
| Other contacts/patient (N) | 0.221 |
| Unknown contact (O) | 0.341 |

1. Sources: Cardol, M. Huisartsenzorg: wat doet de poortwachter (GP-care, what does the gate-keeper), NIVEL, Utrecht, 2004, table 4.2.1, p. 55 ; Van den Berg, M. e.a. De werkbelasting van huisartsen (Workload of GPs), NIVEL, Utrecht, 2004 [4,12]

The figures above include the contacts with GPs only. Contacts with practice assistants and other unknown contacts are omitted. In 2003, 2.88 patient contacts were liable for financial compensation [17].

Table 11. Income components for both years for Dutch GPs in Euros based on fees and workload

| **Income components** | **1995**  **(Euro)** | **2000**  **(Euro)** | **2005**  **(Euro)** |
| --- | --- | --- | --- |
| Patients < 65 years, public health insurance  (H*(1-I)*J*C) |  | 90,381.11 | 109,613.26 |
| Patients > 65 years public health insurance  (H*I*J*D) |  | 15,936.36 | 19,314.45 |
| Public health insurance < 1600 patients  (1600*A) | 89,260.38 |  |  |
| Public health insurance > 1600 patients  (H*(J-1600)*B) | 1,146.12 |  |  |
| Privat patient consultations  (H*(1-J)*K*E) | 27,446.02 | 31,058.25 | 42,862.80 |
| Private patients home visits  (H*(1-J)*L*F) | 4,700.25 | 5,317.99 | 7,339.37 |
| Private patients telephone visits and other contacts (H*(1-J)*(M+N+O)*G) | 2,027.56 | 6,801.90 | 9,387.31 |
|  |  |  |  |
| **Total income including practice costs** | **124,580.33** | **149,495.61** | **188,517.19** |
| Extra income: influenza vaccination | - | 3,498.77 | 4,034.68 |
|  | **1995**  **(pppUS$)** | **2000**  **(pppUS$)** | **2005**  **(pppUS$)** |
| Total income including practice costs in pppUS$ | **137,269.46** | **160,550.11** | **202,061.18** |

Administering influenza vaccinations to high-risk patients resulted in additional income of approximately 3,500 euro. This is calculated as follows: 22.5% of the Dutch population belongs to one of the high risk groups for whom influenza vaccination is recommended [18]. From the high-risk population in 2000, 76% was vaccinated. This results in approximately 434 vaccinations per full-time GP.

Tabel 12. Estimations of practice costs of Dutch GPs and total income after deduction of practice costs

|  | **D&T**  **2000**  **Euro** | **D&T**  **2000**  **Dfl** | **CTG-Zaio**  **2005**  **euro** | CTG-Zaio **2000**  **Euro^1)^** | **CBS**  **2001**  **Euro** |
| --- | --- | --- | --- | --- | --- |
| Housing | 25,890 | 57,055 | 21,884 | 19,349 |  |
| Furnishing | 5,937 | 13,084 | 3,391 | 2,998 |  |
| Medical equipment | 2,276 | 5,015 | 2,214 | 1,958 |  |
| Disposables | 2,433 | 5,361 | 2,594 | 2,294 |  |
| Personel | 41,989 | 92,531 | 32,139 | 28,416 |  |
| Other costs | 25,617 | 56,453 | 18,749 | 16,577 |  |
| Transportation | 4,070 | 8,968 | 2,637 | 2,332 |  |
| Computer, telephone | 14,269 | 31,446 | 12,443 | 11,002 |  |
| Total practice costs excl. locum tenants | 122,481 | 269,913 | 96,051 | 84,926 |  |
|  |  |  |  |  |  |
| Locum tenants | 11,771 | 25,940 | 2,765 | 2,445 |  |
| Total practice costs | 134,252 | 295,853 | 98,816 | 87,370 | 94,042 |
|  |  |  |  |  |  |
| **Practice costs after inflation correction** | **1995** | **2000** | **2005** |  |  |
| Deloitte & Touche (2000) | 120,839 | 134,252 |  |  |  |
| CTG-Zaio (2005) | 78,641 | 87,370 | 98,816 |  |  |
| CBS (2001) | 81,016 | 90,251 | 102,074 |  |  |
| **Practice costs (source CBS) in ppp$** | **89,268** | **94,709** | **107,115** |  |  |
|  |  |  |  |  |  |
|  |  |  |  |  |  |
| Total income excl. practice costs (euro) | 47,548.90 | 62,742.94 | 90,477.49 |  |  |
|  | **1995** | **2000** | **2005** |  |  |
| **Total income excl. practice costs in ppp$** | **52,391.99** | **65,841.55** | **94,945.79** |  |  |

^1)^ Based on data for 2005, corrected for inflation

Sources : D&T: Deloitte and Touche

CTG-Zaio: *College Tarieven Gezondheidszorg/Zorgauthoriteit in oprichting* (Central Body for Fees in Health Care)

CBS: Central Bureau of Statistics

The practice costs for individual solo practices using the CBS-data are calculated as follows: total costs/(percentage solo practices*(total # practices- # practices including farmacies)) =256,000,000/(0.644*(4750-523)). Practice costs include civil liability insurances and legal expenses insurances. Disability insurances and superannuation contributions are paid from the annual income.

In the calculations we used the CBS-estimate for practice costs.

### Sweden

*Type of remuneration*

In Sweden, the GPs (*Husläkare*) used to receive a salary and are employed by the county councils. However, in the past decade, the remuneration system became differentiated among the counties. Nowadays there are as many health care systems as there are counties and with each is own mix of salary, capitation and fee-for-services for the remuneration of their GPs. For 2005, we therefore did not establish a nation wide remuneration system.

*Sources and calculation of revenue and income*

The data originate from the Swedish Medical Association’s statistics (*Sveriges läkarförbund*) and represent the average income of a GP.

Table 13. Estimation of income of Swedish GP

|  | **1995** |  | **2000** |  | **2005** |  |
| --- | --- | --- | --- | --- | --- | --- |
|  | **SEK** | **pppUS$** | **SEK** | **pppUS$** | **SEK** | **pppUS$** |
| Income incl practice costs | 418,000 | **47,500** | 525,000 | **54,124** | 640,000 | **66,667** |
| Practice costs | - | **-** | - | **-** | - | **-** |
| Income excl practice costs | 418,000 | **47,500** | 525,000 | **54,124** | 640,000 | **66,667** |

### United Kingdom

*Type of remuneration*

The income of physicians in the United Kingdom could be characterised as a mix of a kind of salary (a basic allowance) and capitation fees. Before 2004, the income of physicians in the United Kingdom was based on a basic allowance, increased with allowances based on, amongst others, number of listed patients, patient characteristics (age, chronic conditions, living in deprived areas) and some types of services rendered. There was a slight gradient in income due to seniority (depending on the number of years a GP is registered). After 2004, the income became practice based and was related to characteristics of the patients on the list and as such can be characterized as a capitation fee system.

*Establishment of tariffs and income*

For 1995 and 2000 the capitation fee was based on the recommendations of the Review Body on Doctors’ and Dentists’ Remuneration. This independent organisation was established in 1971. Its role was to make recommendations to the Prime Minister and the Secretary of State for Health on the remuneration of doctors and dentists taking any part in the National Health Service. In 2005, the so-called General Medical Services contract was introduced, a three-year contract that is negotiated between the British Medical Association and the Ministry of Health.

*Detailed description of (changes in) the remuneration system*

The income of physicians in the United Kingdom was derived from capitation fees that for 1995 and 2000 were based on the recommendations of the Review Body on Doctors’ and Dentists’ Remuneration. The so-called basic intended income of a GP in 1995 should be £43,165. This amount was calculated based on a complex set of allowances like basic allowances based on the patients on the list, patient’s age specific allowances, deprivation payments for listed patients resident in a deprived area, out-of-hour coverage and night- and emergency visits, extra payments for seniority, and several preventive activities. The intended amount could differ from the actual realized amount after one year, therefore, corrections were made for over- or underpayments in the year(s) before. For instance, in 1995 this correction was £ -217. The actual (average) remuneration in 1995 was £ 42,291. In the year 2000 the intended average remuneration was £ 54,220 and the net actual pay was £ 56,406. In our study we used the actual remuneration (instead of the pre-calculated intended remuneration). The practice costs, including cost of a motor car, telephone, ancillary staff employment and practice rent were estimated by the Review body on Doctors' and Dentists' Remuneration for 1995 and 2000 [19,20]. In 2005 the practice costs were negotiated in the General Medical Services Contract [21].

In 2004, the payment system changed from GP based to practice based, with a global budget. The practice payment is based on characteristics of the patient population on the list. Adjustments are made for age and gender distribution, morbidity and mortality, number of nursing home residents, list turnover and additional costs due to cost of living in the area and rurality. In 2005 an average practice with 5500 patients and about three full time GPs, with average population need and service costs received a global sum payment of £ 305,000, which is about £ 54 per patient [21]. Additional income can be earned when certain quality requirements were met. There were four domains for quality improvement: the clinical domain (with emphasis on certain diseases), the organisational domain (amongst others: information, communication, education and practice management), the additional services domain (cervical screening, child health surveillance, maternity services and contraceptive services) and finally the patient experience domain which consists of how services are provided and the involvement of patients in service development plans. The degree in which a quality indicator is met is awarded with points. Each point represents the value of £ 120 in 2005 for an average list size. The maximum number of point to be scored is 1050. Under the new contract GPs are generously compensated for investments in computing equipment.

In the UK GPs receive a 5% additional contribution for their pension and they contribute 6% out of their own income. (The superannuation contributions are not included in the UK GP-income figures, including these figures would increase the income with £ 6,234) [22].

Under the new contract GPs have the option to opt out of providing night and weekend care. About 90% of the GPs took up this option. [23].

There is a slight gradient in income due to seniority (depending on the number of years a GP is registered).

*Sources and calculation of revenue and income*

The income of 1995 and 2000 is based on publications of the Review Body on Doctors’ and Dentists’ Remuneration [20] .The income of the year 2004/2005 is derived from the GP Earnings and Expenses Enquiry 2004/2005 [22]. This refers to average GP income.

Table 14. Estimation of income of GP in the United Kingdom

| **UK** | **1995^1)^** |  | **2000^1)^** |  | **2005^2)^** |  |
| --- | --- | --- | --- | --- | --- | --- |
|  | **£** | **pppUS$** | **£** | **pppUS$** | **£** | **pppUS$** |
| Income incl practice costs | 63,991 | **106,652** | 79,386 | **113,409** | 230,097 | 383,495 |
| Practice costs | 21,700 | **36,167** | 22,980 | **32,829** | 129,927 | 216,545 |
| Income excl practice costs | 42,291 | **70,485** | 56,406 | **80,580** | 100,170 | 166,950 |

^1)^ Review body on Doctors' and Dentists' Remuneration. Thirty-first report 2002. London, 2002 [20]

^2)^ GP Earnings and Expenses Enquiry 2004/05 Final Report, The Information Centre, Government Statistical Service, 2006 [22]

### GP-INCOME: THIS STUDY COMPARED WITH OTHER STUDIES

Two other international studies have been published in the last five years. The first study is the Stethos study, which is based on questionnaires sent to individual GPs in several countries in 2004/2005[24]. Three countries overlapped with our study. The data in the Stethos study have not been corrected for differences in consumer price levels. The second data source is the OECD [25], who collected data on physician remuneration in 13 European countries. These data were corrected for differences in consumer price levels.

[Table 15 about here]

Because of the differences in currency and correction factors, the comparison between the studies will be based on ranking of the countries. For both the Stethos study and the OECD study, we see that there is considerable difference in outcome. For comparison between OECD and this study, in 2000 enough observations were available for statistical analysis. To improve comparability of the data, the ratio to GDP per capita was used for calculation of the correlations. The results indicate a reasonable correlation between the two studies (Pearson’s r = 0.81, p= 0.02). Striking is the structural difference in ratio between the OECD data and our study. The OECD ratios are consequently higher compared to our findings, except for Germany. Especially for most of the non-salary countries, the difference is considerable (France (0.76), Belgium (1.02), Netherlands (1.42)). The ranking in the Stethos study is comparable to our study, but in our study the range between lowest and highest income is much larger. For 2005, the ranking of the GP-income ratio to GDP per capita is quite comparable between OECD and this study. The income for the UK in our study is much higher, this is probably due to the high rise in income after the introduction of the new remuneration system in the UK in 2004-2005. The income for Belgium is much lower in our study, which is probably due to the high estimate of the practice costs in our study.

Tabel 15. Results of two other data sources of income of GPs compared with this study

|  | **Annual income 2000** | | **Ratio GP-income/GDP per capita**  **2000** | | **Difference** | **Annual income**  **2005** | | | **Ratio GP-income/GDP per capita**  **2005** | | **Difference** |
| --- | --- | --- | --- | --- | --- | --- | --- | --- | --- | --- | --- |
| **Country** | **OECD [25]^1)^**  **pppUS$** | **This study**  **pppUS$** | **OECD [25]^2)^**  **(A)** | **This study**  **(B)** | **(A-B)** | **Stethos [24]**  **(Euro)** | **OECD^3)^**  **pppUS$** | **This study**  **pppUS$** | **OECD^3)^**  **(C)** | **This study**  **(D)** | **(C-D)** |
| Belgium | 52,099 | 25,602 | 1.98 | 0.96 | 1.02 | - | 74,148 | 33,769 | 2.3 | 1.02 | 1.28 |
| Denmark | 92,666 | 85,362 | 3.25 | 2.96 | 0.29 | - | - | 108,295 |  | 3.17 |  |
| Finland | 51,654 | 47,213 | 2.01 | 1.83 | 0.18 | - | 60,164 | 69,725 | 1.9 | 2.26 | -0.36 |
| France | 76,889 | 53,889 | 2.78 | 2.02 | 0.76 | 60,450 | - | 71,000 |  | 2.34 |  |
| Germany | 86,719 | 69,325 | 3.61 | 3.77 | -0.16 | 70,893 | 111,872 | 111,694 | 3.7 | 3.63 | 0.07 |
| Netherlands | 113,147 | 65,842 | 3.73 | 2.31 | 1.42 | - | 123,070 | 94,946 | 3.5 | 2.99 | 0.51 |
| Sweden | 62,468 | 54,124 | 2.22 | 1.99 | 0.23 | - | - |  |  | - |  |
| UK | 85,731 | 80,580 | 3.41 | 3.15 | 0.26 | 71,825 | 120,550 | 166,950 | 3.8 | 5.08 | -1.28 |

.^1)^ The data refer to the year 2000, except for Germany (1999), France (2001), Sweden (2002) and the Netherlands (2003).

^2)^ GDP per capita for the year of origin of the data

^3)^ The data refer to the year 2004 for Belgium, Gemany and UK, to the year 2005 for Finland and Netherlands, source: OECD health data files 2007
