# Supplementary material for: Income development of General Practitioners in eight European countries from 1975 to 2005
Source: BMC Health Serv Res. 2009 Feb 9;9:26. doi: 10.1186/1472-6963-9-26 (PMC2670288; doi:10.1186/1472-6963-9-26)
Supplement: Additional file 2 — Overview of annual GP income for the years 1995, 2000 and 2005 in pppUS$1), countries in alphabetical order. [file 1472-6963-9-26-S2.doc]

Table 2: Overview of annual GP income for the years 1995, 2000 and 2005 in pppUS$^1)^, countries in alphabetical order

|  | **1995** |  |  |  |  | **2000** |  |  |  |  | **2005** |  |  |  |  |
| --- | --- | --- | --- | --- | --- | --- | --- | --- | --- | --- | --- | --- | --- | --- | --- |
|  | **Revenues** | **Practice costs** | | **Income (without practice costs)** | **Rank** | **Revenues** | **Practice costs** | | **Income (without practice costs)** | **Rank** | **Revenues** | **Practice costs** | | **Income (without practice costs)** | **Rank** |
| **Country** |  | **pppUS$** | **Share in total revenues** |  |  |  | **pppUS$** | **Share in total revenues** |  |  |  | **pppUS$** | **Share in total revenues** |  |  |
| **Belgium** | 62,794 | 43,580 | 69% | 19,214 | 7 | 72,312 | 46,709 | 65% | 25,602 | 8 | 85,710 | 51,941 | 61% | 33,769 | 8 |
| **Denmark** | 143,953 | 69,214 | 48% | 74,739 | 2 | 155,471 | 70,109 | 45% | 85,362 | 2 | 206,835 | 98,541 | 48% | 108,295 | 3 |
| **Finland** | -- | -- |  | -- |  | -- | -- | -- | 47,213 | 7 | -- | -- | -- | 69,725 | 6 |
| **France** | 95,168 | 40,863 | 43% | 54,305 | 4 | 100,311 | 46,422 | 46% | 53,889 | 6 | 130,778 | 59,778 | 46% | 71,000 | 5 |
| **Germany** | 190,138 | 109,973 | 58% | 80,164 | 1 | 210,171 | 113,846 | 54% | 96,325 | 1 | 236,300 | 124,606 | 53% | 111,694 | 2 |
| **Nether­lands** | 141,659 | 89,268 | 63% | 52,392 | 5 | 160,550 | 94,709 | 59% | 65,842 | 4 | 218,514 | 107,115 | 49% | 105,098 | 4 |
| **Sweden** | -- | -- | -- | 47,500 | 6 | --- | -- | -- | 54,124 | 5 | -- | -- | -- | 66,667 | 7 |
| **UK** | 63,991 | 36,167 | 57% | 70,485 | 3 | 113,409 | 32,829 | 29% | 80,580 | 2 | 383,495 | 216,545 | 56% | 166,950 | 1 |

^1)^ Please note that the income figures are provided in pppUS$ in order to make the figures comparable among the countries. Since the value of one pppUS$ in most cases is less than the local currency (for example Euros and Pounds), the income figures are higher compared to the same figures expressed in local currencies. For instance, the income in the UK in 2004/5 was £100,170. The conversion rate towards pppUS$ was 1.640689 (World development indicators 2006, The World Bank, Washington DC, USA, Table 4.14), resulting in an annual income of 166,950 in pppUS$.
